# Data-intensive analysis of HIV mutations

**DOI:** 10.1186/s12859-015-0452-0

**Published:** 2015-02-05

**Authors:** Mina Cintho Ozahata, Ester Cerdeira Sabino, Ricardo Sobhie Diaz, Roberto M Cesar-, João Eduardo Ferreira

**Affiliations:** Department of Computer Science - DCC, University of São Paulo, Rua do Matão, 1010, CEP 05508-090 São Paulo, SP Brazil; Sangue Foundation, Health State Secretary, Department of Molecular Biology, Serology Division, Av Dr Enéas de Carvalho Aguiar, Cerqueira Cesar, CEP 05403-000 São Paulo, 155 SP Brazil; Federal University of São Paulo, Rua Pedro de Toledo, São Paulo, 669, CEP 04039-032 SP Brazil

**Keywords:** HIV, Mutation, Cluster

## Abstract

**Background:**

In this study, clustering was performed using a bitmap representation of HIV reverse transcriptase and protease sequences, to produce an unsupervised classification of HIV sequences. The classification will aid our understanding of the interactions between mutations and drug resistance. 10,229 HIV genomic sequences from the protease and reverse transcriptase regions of the pol gene and antiretroviral resistant related mutations represented in an 82-dimensional binary vector space were analyzed.

**Results:**

A new cluster representation was proposed using an image inspired by microarray data, such that the rows in the image represented the protein sequences from the genotype data and the columns represented presence or absence of mutations in each protein position.The visualization of the clusters showed that some mutations frequently occur together and are probably related to an epistatic phenomenon.

**Conclusion:**

We described a methodology based on the application of a pattern recognition algorithm using binary data to suggest clusters of mutations that can easily be discriminated by cluster viewing schemes.

## Background

The human immunodeficiency virus (HIV) shows extensive genetic variability that helps the selection of drug resistance mutations in response to antiretroviral therapy. Hence, it is important to understand the relationship between HIV genotype and phenotype (i.e., drug resistance) to increase the probability of treatment success.

To infer antiretroviral resistance, look-up tables [1,2] and rule-based systems [3,4] were developed by different groups to infer phenotypic resistance based on HIV genomic sequences from infected patients that failed on antiretroviral therapy. In Brazil, a look-up table [2] was developed and used by the Brazilian Ministry of Health AIDS program to help the decision-making process for antiretroviral salvage therapy (http://algoritmo.aids.gov.br/).

In Brazil, patients who fail on antiretroviral therapy receive genotype tests for antiretroviral resistance throughout a network of laboratories [5]. This collection of HIV genomic sequences represents the variability of the HIV population in this country. With this extensive amount of data, questions arise as to whether it is possible to classify the sequences, based on the occurrences of resistance-related mutations in the different amino acid positions, and whether it is possible to achieve a classification that can express current knowledge of the relationship between mutations and drug resistance.

One possible way to answer these questions is to apply clustering algorithms on reverse transcriptase and protease sequences, to obtain clusters containing sequences that are similar. This similarity among the sequences may reveal some of the relationships among the mutations related to antiretroviral resistance.

Nonetheless, extraction of a simple and compact representation of the dataset is complex because of the number and size of sequences. The clusters thus generated may provide a representation that contributes to the understanding of the classification and the relationships between mutations.

**Figure 1:**
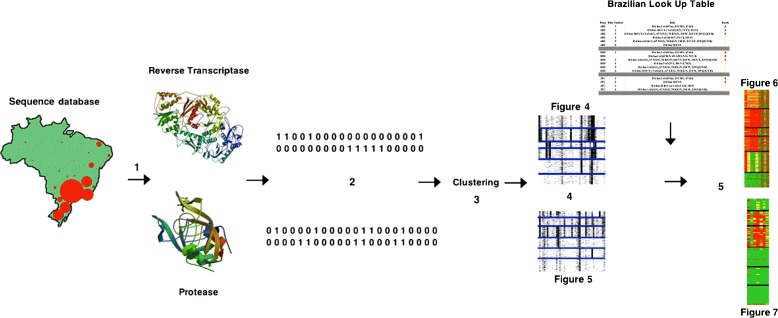
**Pipeline summarizing the proposed framework.** **1)** Protease and reverse transcriptase sequences were gathered from patients from all over Brazil, **2)** binarization of the sequences, **3)** clustering of the mutations, **4)** characterization of the clusters and **5)** comparison with the Brazilian look-up-table predictions.

In the present study, a pipeline (see Figure 1) was introduced to represent clusters inspired by microarray data, in which extensive amounts of data are available. Microarray data were used as inspiration because such applications typically contain large volumes of information on gene patterns from thousands of genes at once. Thus, clusters were represented in an image corresponding to a matrix, such that the rows in the image represented each protein sequence and the columns indicated the presence or absence of resistance-related mutations. This image enabled us to summarize the dataset without losing any information about clustering, permitting the observation of important characteristics of each cluster and enabling cluster comparison, thus providing insights into the data.

Previous studies have attempted to identify common protease and reverse transcriptase mutation patterns [6-15] (as shown in Tables 1, 2 and 3). However, many previous works search only for pairs of mutations, not being able to find larger mutation patterns, which are known to exist [11,16-21]. Furthermore, frequently, only subtype B virus sequences are used, and mutations occur with different probabilities in the different subtypes [22]. Also, in some of the previous works a small number of protein positions are used. Consequently, not all mutation patterns in the data are found and it is more difficult to compare results. Finally, small datasets used in some of the related works do not represent all of the virus population variability, also missing mutation patterns. Therefore, there is no clear consensus on which are the important mutation patterns that arise in the protein sequences.

**Table 1:** **Related works**

**Author**	**Proteins**	**Drugs**	**Protein positions**	**Mutation patterns**	**Number of sequences**	**Method**
Liu et al. 2008 [7]	Protease	PI	PR1 to PR99	(PR30 PR75 PR88),	7758+8761 (Subtype B and non-Subtype B)	k-way clustering
				(PR1–PR9 PR12–PR15		
				PR17 PR19 PR20 PR22		
				PR25 PR26 PR28		
				PR31 PR35–PR42		
				PR45 PR49 PR52)		
				(PR56 PR57 PR59		
				PR61 PR65 PR68–PR70		
				PR77 PR83 PR87		
				PR89 PR96–PR99)		
				(PR1 PR2 PR9 PR26 PR30		
				PR40 PR45 PR56		
				PR59 PR75 PR81 PR88 PR98)		
				(PR13–PR15 PR20 PR35–PR38		
				PR41 PR42 PR49 PR57		
				PR69 PR70 PR77 PR83 PR89)		
				(PR10 PR23 PR24		
				PR27 PR32–PR34 PR43		
				PR46–PR48 PR50 PR53–PR55		
				PR58 PR71 PR76 PR80 PR82)		
				(PR30 PR75 PR88)		
				(PR1 PR2 PR9 PR26		
				PR40 PR45 PR59 PR87 PR98)		
				(PR13–PR15 PR20 PR35–PR38		
				PR41 PR49 PR57 PR69		
				PR70 PR77 PR83 PR89)		
				(PR10 PR23 PR24		
				PR27 PR32–PR34		
				PR42 PR43 PR46–PR48		
				PR50 PR53–PR55		
				PR58 PR71PR76 PR80 PR82)		
Reuman et al. 2010 [8]	Reverse transcriptase	NNRTI	RT90, RT94, RT98,	(RT101,RT181,RT190)	13039	Jaccard similarity
			RT100, RT101, RT102	(RT103,RT181,RT190)	(10504 Subtype B,	coefficient,
			RT103, RT105, RT106,	(RT108,RT181,RT221)	747 Subtype C,	Holm’s correction,
			RT108, RT138,	(RT98,RT181,RT190)	363 (CRF) 01_AE,	Poissoness plot
			RT139, RT178, RT179,	(RT181,RT190,RT221)	210 Subtype A,	
			RT181, RT188,	(RT103,RT181,RT221)	320 CRF 02_AG,	
			RT190, RT221, RT223,	(RT103,RT108,RT221)	895 others)	
			RT225, RT227,	(RT101,RT108,RT181)		
			RT230, RT232,	(RT101,RT108,RT190)		
			RT234, RT236,	(RT103,RT108,RT181)		
			RT237, RT238,	(RT108,RT190,RT221)		
			RT241, RT242, RT318	(RT98,RT108,RT181)		
				(RT98,RT101,RT190)		
				(RT98,RT101,RT181)		
				(RT101,RT181,RT190)		
				(RT101,RT181,RT221)		
				(RT98,RT103,RT108)		
				(RT101,RT181,RT190)		
				(RT108,RT181,RT190)		
				(RT98,RT103,RT181)		
Wu et al. 2003 [10]	Protease	PI	PR1 to PR99	(PR10 PR63	2244 (Subtype B)	binomial correlation
				PR71 PR73 PR90)		coefficients, pca
				(PR10 PR63		
				PR71 PR90 PR93)		
				(PR10 PR62		
				PR63 PR90 PR93)		
				(PR10 PR62		
				PR63 PR73 PR90)		
				(PR10 PR20		
				PR71 PR73 PR90)		
				(PR10 PR20		
				PR62 PR73 PR90)		
				(PR10 PR46		
				PR71 PR90 PR93)		
				(PR10 (PR30)		
				PR73 PR84 PR90)		
				(PR10 (PR30)		
				PR46 PR84 PR90)		
				(PR10 PR71 PR73 PR84 PR90)		
				(PR10 PR46 PR71 PR84 PR90)		
				(PR10 PR24 PR46		
				PR10 PR46 PR90)		
				(PR10 (PR30)		
				PR46 PR54 PR82)		
				(PR10 PR48 PR54 PR82)		
				(PR10 PR24		
				PR46 PR54 PR82)		
				(PR32 PR46 PR82)		
				(PR10 PR46 PR53		
				PR54 PR71 PR82)		
				(PR30 (PR82) PR88)		
				(PR13 PR30 PR88)		
				(PR30 PR75 PR88)		
				(PR10 PR46		
				PR63 PR71 PR93)		
				(PR20 PR36 PR54)		
				(PR10 PR20 PR54 PR71)		
				(PR63 (PR64) PR71)		
				(PR10 PR77 PR93)		
				(PR20 PR36 PR62)		
				(PR20 PR35 PR36 (PR77))		
				(PR15 PR20 PR36 (PR77))		
				(PR10 PR24 PR89)		
				(PR10 PR20 PR73)		
				(PR10 PR73 PR77)		

Protease positions are represented by the prefix PR and reverse transcriptase positions by the prefix RT.

**Table 2:** **Related works**

**Author**	**Proteins**	**Drugs**	**Protein positions**	**Mutation patterns**	**Number of sequences**	**Method**
Rhee et al. 2004 [9]	Protease	PI,	PR24, PR30, PR32,	(PR30,PR88) (PR46,PR90)	2795	
	and Reverse	NRTI,	PR46, PR47, PR48,	(PR73,PR90)	(27 Subtype C,	
	transcriptase	NNRTI	PR50, PR53, PR54,	(PR54,PR82,PR90)	15 Subtype A,	
			PR73, PR82, PR84,	(PR24,PR46,PR54,PR82)	7 Subtype D,	
			PR88, PR90	(PR73,PR84,PR90)	2746 Subtype B)	
			RT41, RT44, RT62,	(PR46,PR54,PR82,PR90)		
			RT65, RT67, RT69,	(PR84,PR90) (PR46,PR88)		
			RT70, RT74, RT115,	(PR46,PR73,PR90) (PR54,PR82)		
			RT116, RT118, RT151,	(PR46,PR84,PR90)		
			RT184, RT210,	(PR46,PR54,PR82,PR90)		
			RT215, RT219	(PR46,PR73,PR84 PR90)		
				(PR30,PR88,PR90)		
				(PR48,PR54,PR82),		
				(PR32,PR46,PR82,PR90)		
				(PR24,PR46,PR54,PR82)		
				(PR53,PR54,PR82,PR90)		
				(PR24,PR46,PR82) (PR46,PR82)		
				(PR46,PR90) (PR30,PR46,PR88)		
				(RT41, RT184, RT215)		
				(RT41, RT184, RT210)		
				(RT41, RT215)		
				(RT67, RT70, RT184, RT219)		
				(RT70, RT184)		
				(RT41, RT210, RT215)		
				(RT184, RT215)		
				(RT41, RT118, RT184)		
				(RT210, RT215)		
				(RT41, RT67, RT118, RT210, RT215)		
				(RT74, RT184)		
				(RT67, RT70, RT184)		
				(RT67, RT69, RT70, RT184, RT219)		
				(RT41, RT67, RT184,		
				RT210, RT215)		
				(RT41, RT184)		
				(RT62, RT184)		
				(RT41, RT44, RT67, RT118)		
				(RT184, RT210, RT215)		
				(RT67, RT70, RT184, RT215, RT219)		
				(RT67, RT70, RT219)		
				(RT67, RT70)		
				(RT41, RT184, RT215)		
				(RT41, RT118, RT210, RT215)		
				(RT41, RT67, RT210, RT215)		
				(RT69, RT70)		
				(RT41, RT44, RT67, RT118,		
				RT210, RT215)		
				(RT41, RT74, RT184, RT215, RT69)		
				(RT103 RT181)		
				(RT100 RT103)(RT103 RT108)		
				(RT101 RT190)		
				(RT103 RT225)		
				(RT103 RT181 RT190)		
				(RT103 RT190)		
				(RT181 RT190)		
				(RT103 RT238)(RT101 RT103)		
				(RT108 RT181)		
				(RT101 RT181 RT190)		
				(RT98 RT103)		
				(RT103 RT108 RT181)		
				(RT103 RT188)(RT103 RT230)		
Gonzales et al. 2003 [11]	Protease	PI,	RT41, RT62, RT65,	(RT41,RT184,RT215)	487	Fisher’s
	and Reverse	NRTI,	RT67, RT69, RT70,	(RT41,RT184,RT210,RT215)	(Subtype B)	exact
	transcriptase	NNRTI	RT74, RT75, RT77,	(RT67,RT70,RT215,RT219)		test,
			RT115, RT116, RT151,	(RT41,RT67,RT69,RT210,RT215)		Benjamini-
			RT184, RT210,	(RT41,RT67,RT184,RT210,		Hochberg,
			RT215, and RT219	RT215,RT219)		K-medoids
			PR24, PR30, PR32,	(RT41,RT67,RT69,RT70,		
			PR46, PR47, PR48,	RT184,RT215,RT219)		
			PR50, PR53, PR54,	(RT65,RT70,RT75,RT77,RT115„		
			PR73, PR88, PR82,	RT116,RT151,RT184,RT219)		
			PR84, and PR90	(PR54,PR73,PR84,PR90)		
				(PR46,PR84,PR90)		
				(PR24,PR46,PR54,PR82)		
				(PR46,PR54,PR82,PR90)		
				(PR48,PR,54,PR82)		
Sing et al. 2005 [6]	Reverse	NRTI	RT41, RT43, RT44, RT62,	(RT41, RT210,RT215)	1355	hierarchical
	transcriptase		RT67, RT69, RT70,	(RT67,RT70,RT219)		clustering,
			RT74, RT75, RT77,			Fisher’s
			RT116, RT118, RT151,			exact test
			RT203, RT208,			
			RT210, RT215, RT215,			
			RT218, RT219,			
			RT219, RT223,			
			RT228, RT228			
Brehm et al. 2012 [41]	Reverse	NNRTI		(RT184,RT348)	12	
	transcriptase				(Subtype C)	

Protease positions are represented by the prefix PR and reverse transcriptase positions by the prefix RT.

**Table 3:** **Related works**

**Author**	**Proteins**	**Drugs**	**Protein positions**	**Mutation patterns**	**Number of sequences**	**Method**
Hoffman et al. 2003 [12]	Protease	PI	PR10, PR12, PR13, PR14,	(PR10,PR93) (PR12,PR19)	1179	Mutual
			PR15, PR19, PR20, PR30,	(PR35,PR38)(PR63,PR64)	(Subtype B)	information
			PR32, PR35, PR36, PR37,	(PR37,PR41)(PR62,PR71)		
			PR41, PR46, PR48, PR54,	(PR71,PR77) (PR71,PR93)		
			PR57, PR60, PR62, PR63,	(PR77,PR93)(PR12,PR19)		
			PR64, PR69, PR71, PR72,	(PR15,PR77)(PR20,PR36)		
			PR73, PR77, PR82, PR84,	(PR30,PR88)(PR35,PR36)		
			PR88, PR90, PR93	(PR35,PR37)(PR36,PR62)		
				(PR36,PR77)(PR46,PR82)		
				(PR46,PR84)(PR48,PR54)		
				(PR48,PR82)(PR54,PR82)		
				(PR63,PR64)(PR63,PR90)		
				(PR77,PR93)(PR84,PR90)		
				(PR73,PR90)		
Alteri et al. 2009 [13]	Reverse	PI,	RT41, RT65, RT67,	(RT215,RT41,RT210)	213	Binomial
	transcriptase	NRTI,	RT69, RT70, RT74, RT75,	(RT60,RT103)	(Subtype B)	correlation
		NNRTI	RT77, RT100, RT101,			coefficient,
			RT103, RT106, RT115,			Benjamini-
			RT116, RT151, RT181,			Hochberg
			RT184,RT188, RT190,			method
			RT210, RT215, RT219,			
			RT225, RT230, RT236,			
Doherty et al. 2011 [14]	Protease	PI	PR10, PR24, PR30,	(PR10,PR32,PR33,	398	Optimal
			PR32, PR33, PR43,	PR46,PR47,PR54,		integer
			PR46, PR47, PR48,	PR71,PR73,PR84,PR90)		programming-
			PR50, PR53, PR54,	(PR10,PR33,PR43,PR46,		based
			PR71, PR73, PR74,	PR54,PR71,PR82,PR84,PR90)		clustering
			PR76, PR82, PR83,	(PR10,PR24,PR46,		
			PR84, PR88, PR90	PR54,PR71,PR74,PR82)		
				(PR32,PR33,PR46,PR53,		
				PR54,PR71,PR84,PR90)		
				(PR10,PR30,PR32,PR33,PR46,		
				PR54,PR71,PR84,PR88,PR90)		
				(PR10,PR33,PR43,PR46,PR48,		
				PR50,PR54,PR71,PR82)		
				(PR10,PR32,PR46,		
				PR71,PR82,PR84)		
				(PR10,PR46,PR54,PR82,PR90)		
				(PR10,PR48,PR54,PR71,		
				PR73,PR76,PR84,PR90)		
				(PR10,PR24,PR32,PR33, PR43,		
				PR46,PR54,PR71,PR82,PR84)		
				(PR10,PR24,PR30,		
				PR33,PR43,PR53,PR88)		
				(PR10,PR43,PR47,PR48,		
				PR53,PR54,PR71,PR82,PR84)		
				(PR10,PR32,PR46,		
				PR47,PR71,PR82,PR90)		
				(PR10,PR33,PR54,		
				PR73,PR84,PR90)		
				(PR10,PR46,PR71,PR84,PR90)		
				(PR10,PR54,PR71,		
				PR73,PR82,PR90)		
				(PR10,PR32,PR33,		
				PR47,PR71,PR82,PR90)		
				(PR10,PR46,PR54,		
				PR71,PR82,PR90)		
				(PR10,PR24,PR33,PR46,		
				PR54,PR71,PR82)		
				(PR10,PR48,PR54,PR82,PR90)		
				(PR10,PR32,PR43,		
				PR46,PR47,PR82)		
				(PR10,PR54,PR71,PR82)		
				(PR10,PR46,PR47,		
				PR71,PR88,PR90)		
				(PR10,PR33,PR43,PR46,		
				PR50,PR54,PR71,		
				PR73,PR82,PR90)		
				(PR10,PR33,PR46,		
				PR54,PR71,PR88,PR90)		
				(PR10,PR46,PR71,		
				PR74,PR88,PR90)		
				(PR10,PR54,PR74,PR76,PR82)		
				(PR73,PR90)		
				(PR10,PR46,PR90)		
				(PR10,PR71,PR90)		
				(PR10,PR46,PR71)		
				(PR10,PR24,PR46,PR54,PR82)		
Heider et al. 2013 [15]	Reverse	NRTI	RT1 to RT240	(RT41,RT70,	600	Multilabel
	transcriptase			RT210,RT215)	(Subtype B)	classification
				(RT41,RT65,RT67,		
				RT70,RT210,RT215,RT219)		
				(RT65,RT74,RT115)		
				(RT151,RT62,RT69,		
				RT75,RT77,RT116)		
Yahi et al. 1999 [16]	Protease	PI,	PR63, PR77,PR71,	(PR10,PR46) (PR46,PR71)	287	Chi-square
	and	NRTI,	PR10, PR93, PR 36	(PR46,PR90) (PR71,PR82)		or Kendall
	Reverse	NNRTI	PR82, PR46, PR20,	(PR10,PR82) (PR54,PR82)		and
	transcriptase		PR90 and PR54	(PR82,PR90) (PR71,PR90)		Fisher’s
			RT215, RT41, RT67,	(PR10,PR90) (PR46,PR90)		two-tailed
			RT69, RT70, RT184,	(PR54,PR90) (PR77,PR90)		
			RT210 and RT219	(PR82,PR90)		
				(RT41,RT210) (RT67,RT70)		
				(RT69,RT70) (RT70,RT219)		
				(RT41,RT210) (RT184,RT210)		
				(RT210,RT215) (RT70,RT219)		
				(RT67,RT219) (RT69,RT219)		
Melikian et al. 2013 [28]	Reverse	NNRTI		(RT101,RT103,RT106,	1752	Least
	transcriptase			RT181,RT188,RT190)	(1681	angle
				(RT100,RT101,RT103,	Subtype B)	regression
				RT106,RT188,RT190)		(LARS)
				(RT101,RT181,RT190,RT227)		
				(RT100,RT101,RT181,		
				RT190,RT227)		

Protease positions are represented by the prefix PR and reverse transcriptase positions by the prefix RT.

Nonetheless, some patterns have been reported in previous works such as the simultaneous presence of mutations at positions 30 and 88 of the protease [7,9-12,23], selected by nelfinavir [24]. The same applies to thymidine analog mutations (TAMs) in reverse transcriptase, which can be discriminated in TAM1 and TAM2 profiles [11,16-21]. The TAM1 profile presents mutations at codons 41, 210 and 215, whereas TAM2 presents mutation at codons 67, 70, and 219.

Such studies on mutation patterns are important because the co-existence of mutations may result in different antiretroviral resistance profiles. For example, a mutation can restore the fitness decrease from another mutation that confers drug resistance. However, some of the previous studies only investigated pairs of mutations, and most of them only analyzed subtype B HIV-1 sequences. Moreover, previous studies analyzed specific mutation profiles, making it difficult to compare results between different studies. Thus, mutation patterns have not been fully characterized in the protease and reverse transcriptase sequences. Characterization of these patterns may lead to a better understanding of the interactions among these mutations and to classification of the sequences.

In the present study, a large number of codons (38 from reverse transcriptase and 44 from protease, as shown in Table 4) from subtypes B, C and F were clustered, and the sequences were classified according to the mutation patterns. These clusters were compared with clusters reported in other studies.

**Table 4:** **Protease and reverse transcriptase amino acid positions considered in the present study**

	**Protein**	**Position**	**Protein**	**Position**
1	Reverse transcriptase	41	Protease	8
2	Reverse transcriptase	44	Protease	10
3	Reverse transcriptase	50	Protease	11
4	Reverse transcriptase	65	Protease	13
5	Reverse transcriptase	67	Protease	15
6	Reverse transcriptase	69	Protease	16
7	Reverse transcriptase	70	Protease	20
8	Reverse transcriptase	74	Protease	24
9	Reverse transcriptase	75	Protease	30
10	Reverse transcriptase	77	Protease	32
11	Reverse transcriptase	98	Protease	33
12	Reverse transcriptase	100	Protease	34
13	Reverse transcriptase	101	Protease	35
14	Reverse transcriptase	103	Protease	36
15	Reverse transcriptase	106	Protease	41
16	Reverse transcriptase	108	Protease	43
17	Reverse transcriptase	115	Protease	45
18	Reverse transcriptase	116	Protease	46
19	Reverse transcriptase	118	Protease	47
20	Reverse transcriptase	151	Protease	48
21	Reverse transcriptase	157	Protease	50
22	Reverse transcriptase	179	Protease	53
23	Reverse transcriptase	180	Protease	54
24	Reverse transcriptase	181	Protease	57
25	Reverse transcriptase	184	Protease	58
26	Reverse transcriptase	188	Protease	60
27	Reverse transcriptase	190	Protease	62
28	Reverse transcriptase	208	Protease	63
29	Reverse transcriptase	210	Protease	67
30	Reverse transcriptase	211	Protease	69
31	Reverse transcriptase	214	Protease	70
32	Reverse transcriptase	215	Protease	71
33	Reverse transcriptase	219	Protease	73
34	Reverse transcriptase	225	Protease	74
35	Reverse transcriptase	227	Protease	76
36	Reverse transcriptase	230	Protease	77
37	Reverse transcriptase	236	Protease	82
38	Reverse transcriptase	333	Protease	83
39			Protease	84
40			Protease	85
41			Protease	88
42			Protease	89
43			Protease	90
44			Protease	93

### Look up tables and rule-based systems

Based on genotype-phenotype correlation studies on laboratory HIV-1 isolates, genotype-phenotype correlations on clinical isolates and genotype-treatment history correlations [25], some efforts have been made to try to understand the relationship between HIV genotype and phenotype. For example, look-up tables [1,2,26] have been compiled using information from the scientific literature, which has been turned into rules in which the occurrences of mutations, or combinations of mutations, are correlated with drug resistance. In addition to look-up tables, some rule-based systems [3,4] have created scoring systems to calculate the likelihood of therapy failure, which are also based on published data. Look-up tables and rule-based systems are efforts to correlate the set of known mutations with the potential for drug resistance. Both represent current knowledge concerning the relationships between virus genotype and drug resistance and its application. Look-up tables and rule-based systems group mutations into clusters of mutations, thereby predicting the possible result of drug treatment.

### Clustering

Similar to the classifications retrieved from look-up tables and rule-based systems, pattern recognition methods are designed to extract information from data to classify them. In cases where little prior information is available and the decision-maker must make as few assumptions as possible about the data, the clustering technique is useful [27].

By applying clustering algorithms to reverse transcriptase and protease sequences, clusters containing sequences that are similar to each other are obtained. The clusters may contain sequences with similar drug response patterns. Applying clustering algorithms, and comparing the clusters with the classifications from look-up tables will achieve a better understanding of the relationship between genotype and phenotype.

In addition to providing comparisons with look-up tables, clusters also allow hypothesizes regarding the occurrences of mutations to be formed. Therefore, such analysis can show which mutations have higher probability of occurring together and those that may influence each other.

One of the best-known algorithms for clustering is K-means, which is popular because the time complexity is O(n), where n is the number of patterns [27]. The time complexity makes this a good choice when dealing with a large volume of data, which was the case here.

## Methods

### Pipeline

Figure 1 summarizes the methodology used in this work to analyze the protease and reverse transcriptase sequences. First, HIV genomic sequences from patients from 27 Brazilian states were extracted from the national database and binarized according to the presence or absence of mutations. The sequences were clustered and an image was created to represent the clusters. The clusters were characterized given the occurrence of mutations and compared with the prediction of drug resistance from the Brazilian look-up table.

The scripts created for data clustering (step 3) and cluster representation (steps 4 and 5) are available at http://www.ime.usp.br/~mcintho/.

### Sequence representation

In the present study, 10,229 reverse transcriptase and protease sequences from HIV subtype B, 801 from subtype F and 424 from subtype C, were obtained from the national database. These samples were taken in accordance with the ethics standards of the Ethics Committee of the Federal University of São Paulo and with the Helsinki Declaration of 1975, revised in 1983. All biological samples were obtained in full accordance with signed informed consent forms (process number in research ethics committee 1433/09).

The Brazilian Guidelines for Resistance Testing allowed only one genotype testing for each patient at the time the sequences were generated; therefore, duplication of the sequences from the same patient was not expected.

To simplify the representation and comparison of the reverse transcriptase andprotease sequences, bitmap mapping was used. In this technique, if a sequence hadthe same amino acid as the wild-type sequence, it was replaced with the value zeroand when the sequence had a different amino acid, it was replaced by the value1, as previously described (Reuman et al. [8] and Melikian et al. [28]). Thus, the sequences could be interpreted as binary vectors in and 99 dimensional spaces (amino acids from reverse transcriptase and 99 from protease).

When working with patterns of high dimensionality, the “curse of high dimensionality” must be avoided. The “curse of high dimensionality” makes all distances look alike in high dimensional spaces [29] and makes it difficult to evaluate similarity. One way to avoid it is to decrease the dimensionality of the data.

To escape the “curse of high dimensionality”, 38 positions from reverse transcriptase and 44 positions from protease 4 known to be related to drug resistance were analyzed [2,25].

### K-means

In an attempt to classify reverse transcriptase and protease sequences using a pattern recognition algorithm, we applied K-means from the R Project for Statistical Computing [30] to the 10,229 sequences. Sequences were divided according to HIV subtype and genomic region. Thus, K-means was used to search for clusters in the protease and reverse transcriptase sequences from subtypes B, C and F, separately. The algorithm was repeated 10 times for each of the datasets, with random centroids. The value of k, i.e. the number of clusters to be retrieved, ranged from 2 to 16.

### Cluster characterization

One problem that arose from generating the clusters was how to view and interpret them in the domain of HIV mutations, which was caused by the large number of sequences and amino acid positions used in our analysis. Images can be used to solve this problem because they provide an intuitive information visualization tool to support and validate the results, and to formulate and test hypotheses. When the research entails data-intensive analysis, the use of images becomes even more important, because the volume of data makes it difficult to manipulate and visualize the data directly. Thus, images can help in the analysis process and can summarize the data and results.

Therefore, to analyze the clusters, observe whether they followed any mutation patterns and to determine what these patterns might be, images of the clusters were created inspired by microarray data visualization. Binary images (i.e. black and white) represented the binary sequences featured as rows and the amino acid positions as columns: 44 columns for protease and 38 columns for reverse transcriptase. The sequences were grouped according to clusters and separated by blue lines. When a sequence had the value of 1 in an amino acid position, it would be represented by a black pixel, and when it had a value of 0, it would be represented by a white pixel. Six images were created for each value of k, combining the proteins and subtypes.

The black and white pixels were useful for distinguishing the clusters, accentuating differences among them and describing them, as well as for summarizing the information within the sequences and clusters. They also helped to view the amino acid positions that represented and characterized the clusters.

To provide more details about the clusters, histograms were plotted for each cluster, for protease and reverse transcriptase, showing the percentage of sequences in the cluster with mutations in each position. Each bar in the histogram represented an amino acid position and the percentage of sequences in the cluster with a mutation at that position.

To compare the clusters with the look-up table used to interpret the genotypic resistance from the Brazilian algorithm for resistance interpretation, another image was generated. The HIVDAG software [31] was used to create this other image. HIVDAG interprets the rules in the Brazilian look-up table in the context of the sequences and produces a prediction regarding antiretroviral resistance. The software classifies the sequences as resistant (R), intermediate resistant (I) and susceptible (S).

To represent the three possible results, red, yellow and green were used for resistance, intermediate resistance and susceptibility, respectively. Thus, as in the binary figure, the rows featured the protein sequences and the columns were the predictions for drug resistance given by the look-up table for that sequence.

In these colored images, vertical lines presenting a dominant color in each cluster indicated that the sequences in that cluster have the same drug resistance prediction. Clusters that showed red, yellow or green vertical lines in different positions indicated that there was some correspondence between the prediction of the look-up table and the K-means clusters.

## Results and discussion

**Figure 2:**
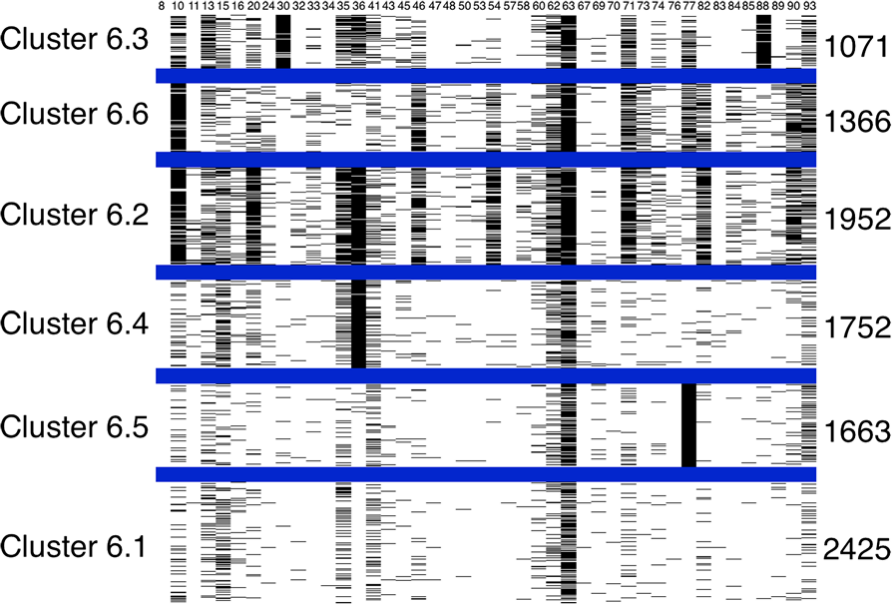
**Black and white figure of kmeans clusters for subtype B sequences of the HIV protease.** The figure displays the different mutation patterns characterizing each subtype B protease cluster. The columns in the figure represent the amino acid positions selected to the clustering and the rows, the protein sequences. Blue lines delimit the six classes, the black pixels represent mutations and the white pixels the absence of mutations. The number identifying each cluster is on the left and the number of the sequences in the cluster on the right.

**Figure 3:**
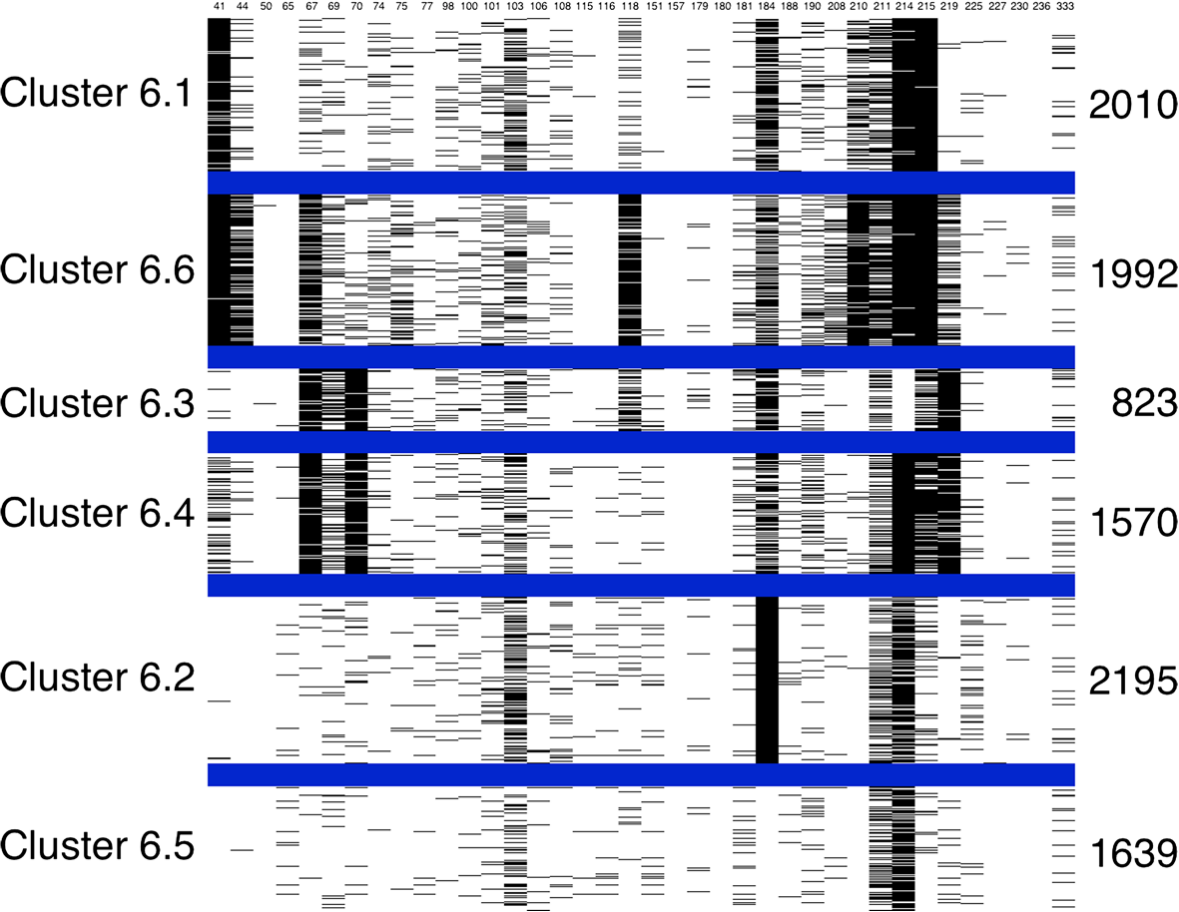
**Black and white figure of kmeans clusters for subtype B sequences of the HIV reverse transcriptase.** The figure displays the different mutation patterns characterizing each subtype B reverse transcriptase cluster. The columns in the figure represent the amino acid positions selected for clustering and the rows represent the protein sequences. Blue lines delimit the six classes, the black pixels represent mutations and the white pixels represent the absence of mutations. The number identifying each cluster is on the left and the number of the sequences in the cluster on the right.

For distinct k values, the sequences were distributed in different clusters; black and white images were created for each combination of subtype, k value and protein. Figures 2 and 3 represent the clusters for subtype B, where k = 6 for protease and reverse transcriptase, respectively. The value of k=6 was chosen because it represents better the current knowledge of mutation occurrence and mutation relationships. For k = 6, both TAM groups and the mutation profile comprising substitutions on protease codons 30 and 88 are represented. Nonetheless, as k values progressed, the clusters were first divided into groups of sequences with many mutations and with few or no mutations. For each increase in the k value, the group with many mutations was repeatedly split, although stability and consistency were maintained.

**Figure 4:**
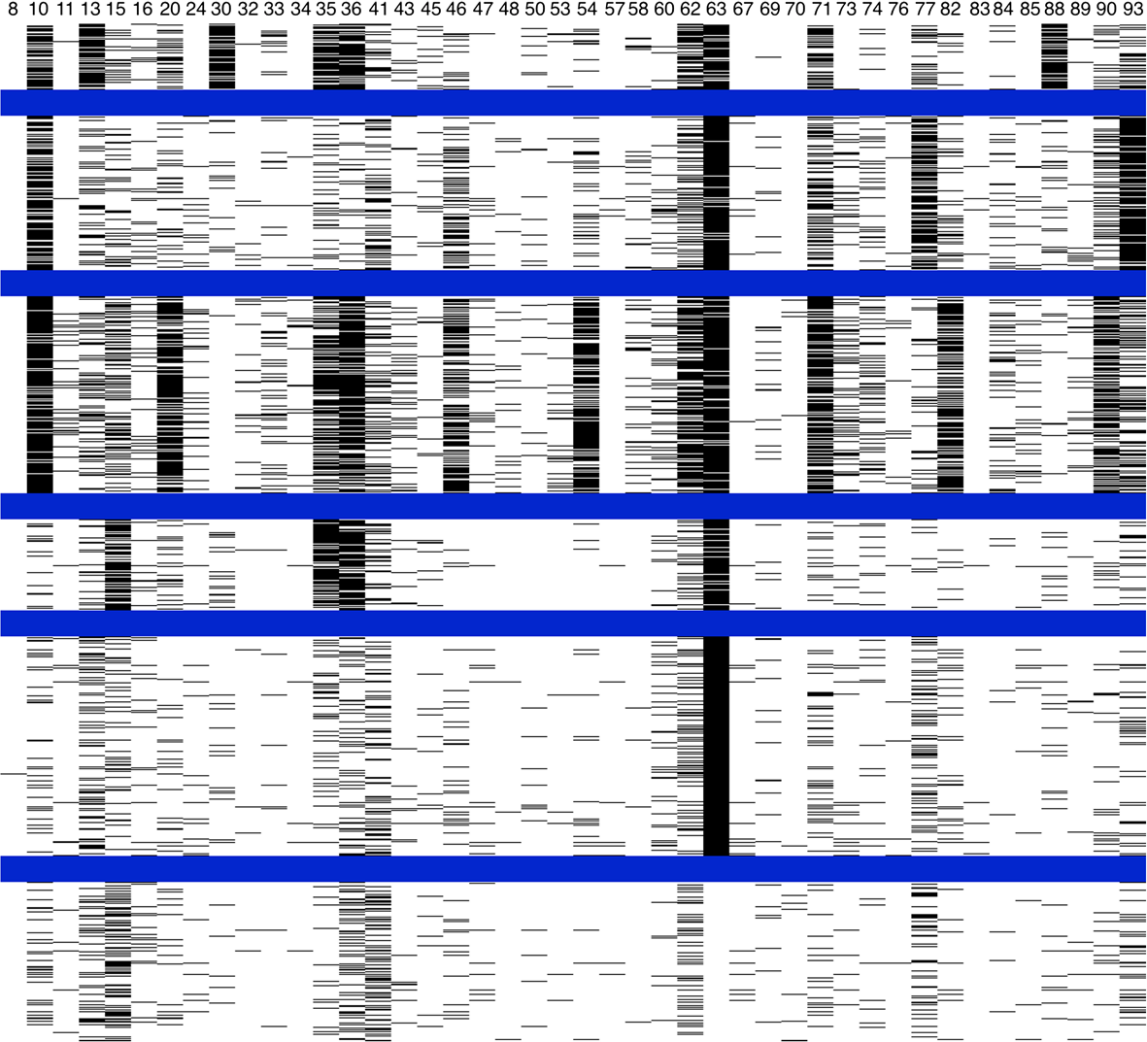
**Black and white figure of k-medoids clusters for subtype B sequences of the HIV protease.** The figure displays the different mutation patterns characterizing each subtype B protease cluster. The columns in the figure represent the amino acid positions selected to the clustering and the rows, the protein sequences. Blue lines delimit the six classes, the black pixels represent mutations and the white pixels the absence of mutations.

**Figure 5:**
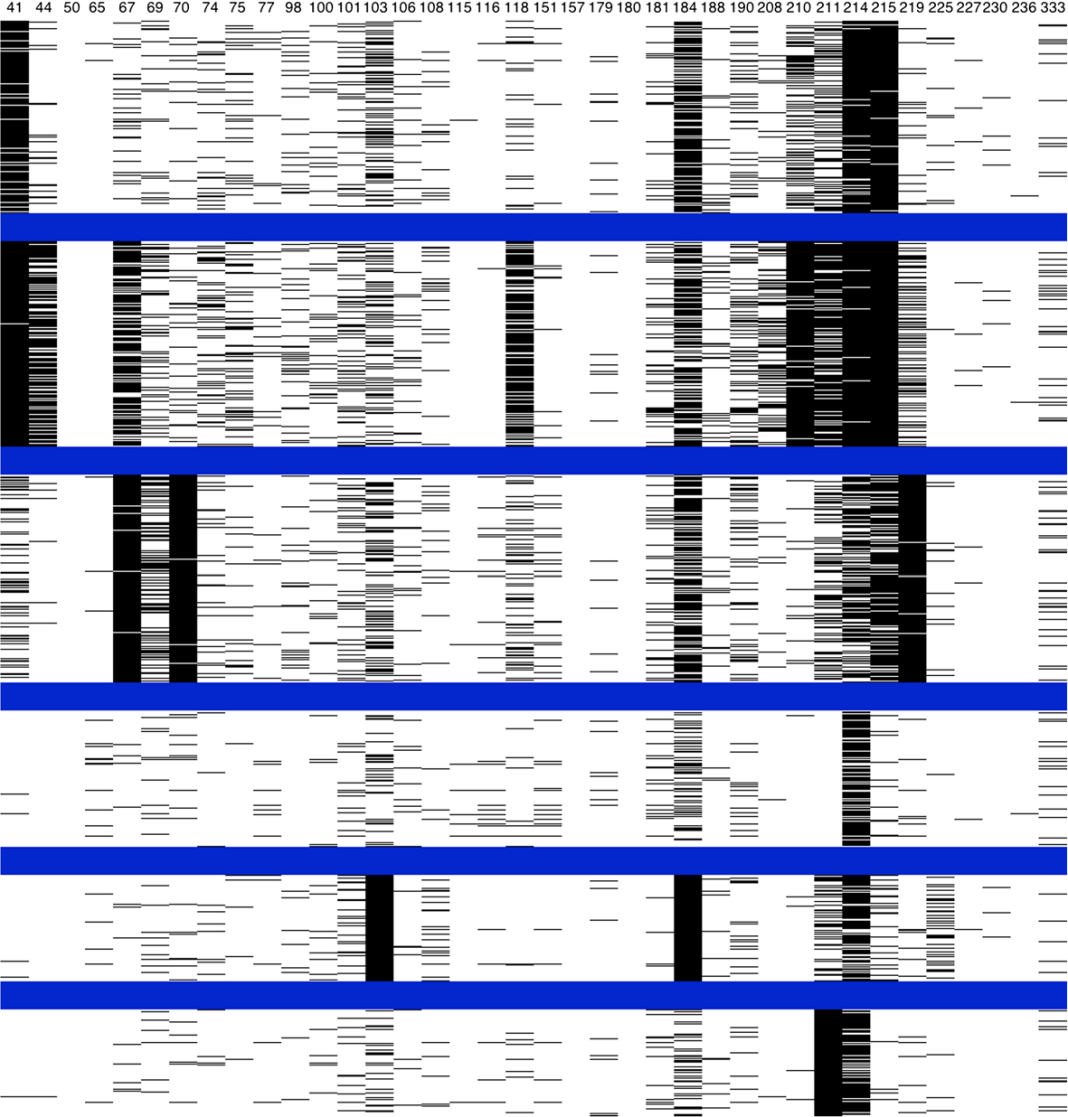
**Black and white figure of k-medoids clusters for subtype B sequences of the HIV reverse transcriptase.** The figure displays the different mutation patterns characterizing each subtype B reverse transcriptase cluster. The columns in the figure represent the amino acid positions selected for clustering and the rows represent the protein sequences. Blue lines delimit the six classes, the black pixels represent mutations and the white pixels represent the absence of mutations.

K-medoids have been used in a previous study [14] for clustering a smaller number of subtype B sequences. In order to evaluate this alternative clustering method, it has been applied to the dataset here described. The K-medoids implementation available at [32] has been adopted and Figures 4 and 5 shows the clustering results. As it can be seen, the results are similar to those shown in Figures 2 and 3, except for clusters B6.4, B6.5 and B6.1 from protease and clusters B6.2 and B6.5 from reverse transcriptase.They contain sequences that are predicted to be susceptible to most of the drugs and do not represent patterns of mutations. This difference is probably because although both algorithms are related, k-medoids represents clusters by the median of cluster points, instead of the mean [33]. But, except for these differences, both methods lead to similar results, which corrobotate our findings.

**Figure 6:**
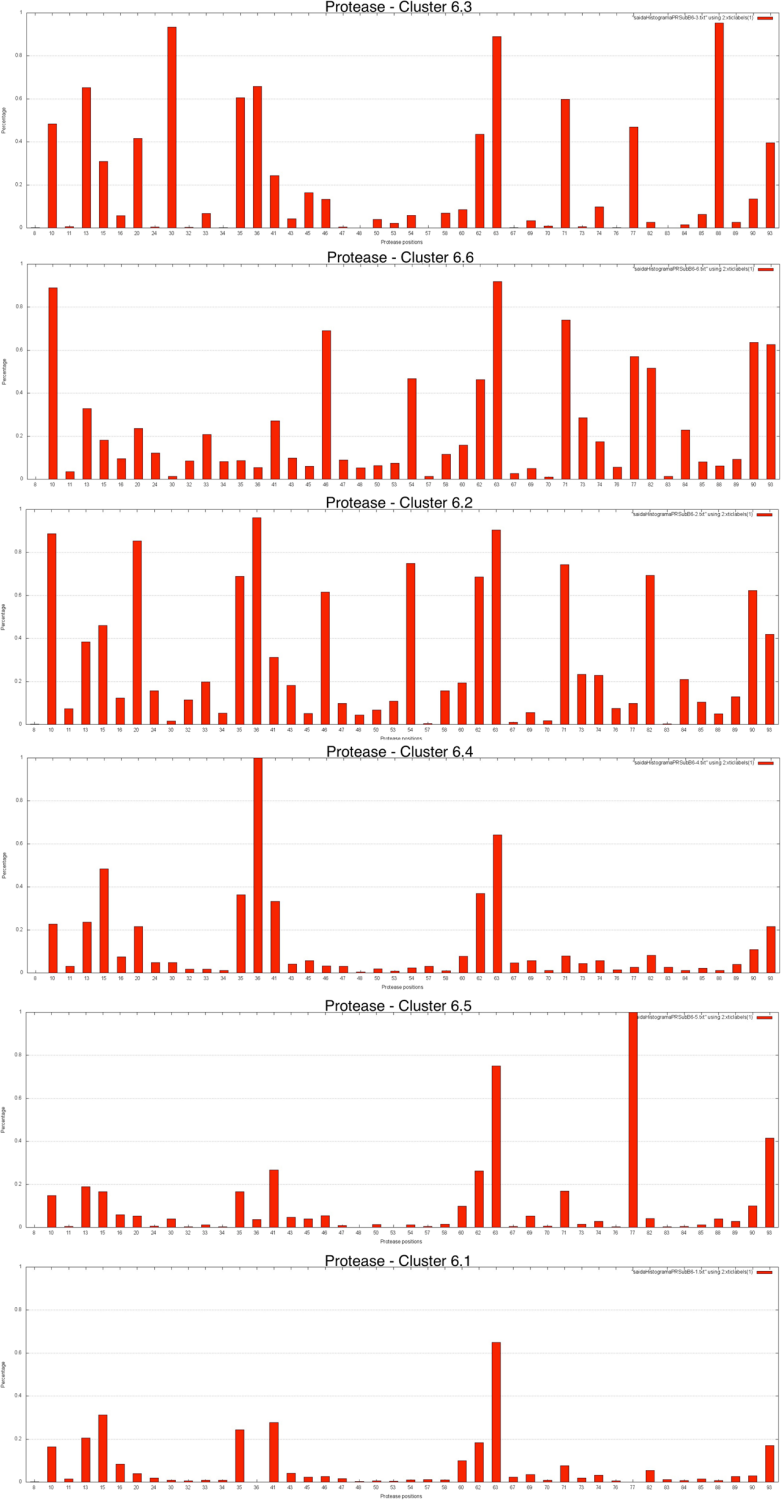
**Histogram showing the frequency of mutations in the protease kmeans clusters.** Histograms containing the frequencies of mutations for each selected amino acid position in protease for each of the six clusters in subtype B at *k*=6. Each histogram represents one cluster found by K-means for *k*=6 in the protease sequences. Each bar in the histogram represents a protein position and the percentage of sequences in the cluster that contain a mutation at that position.

**Figure 7:**
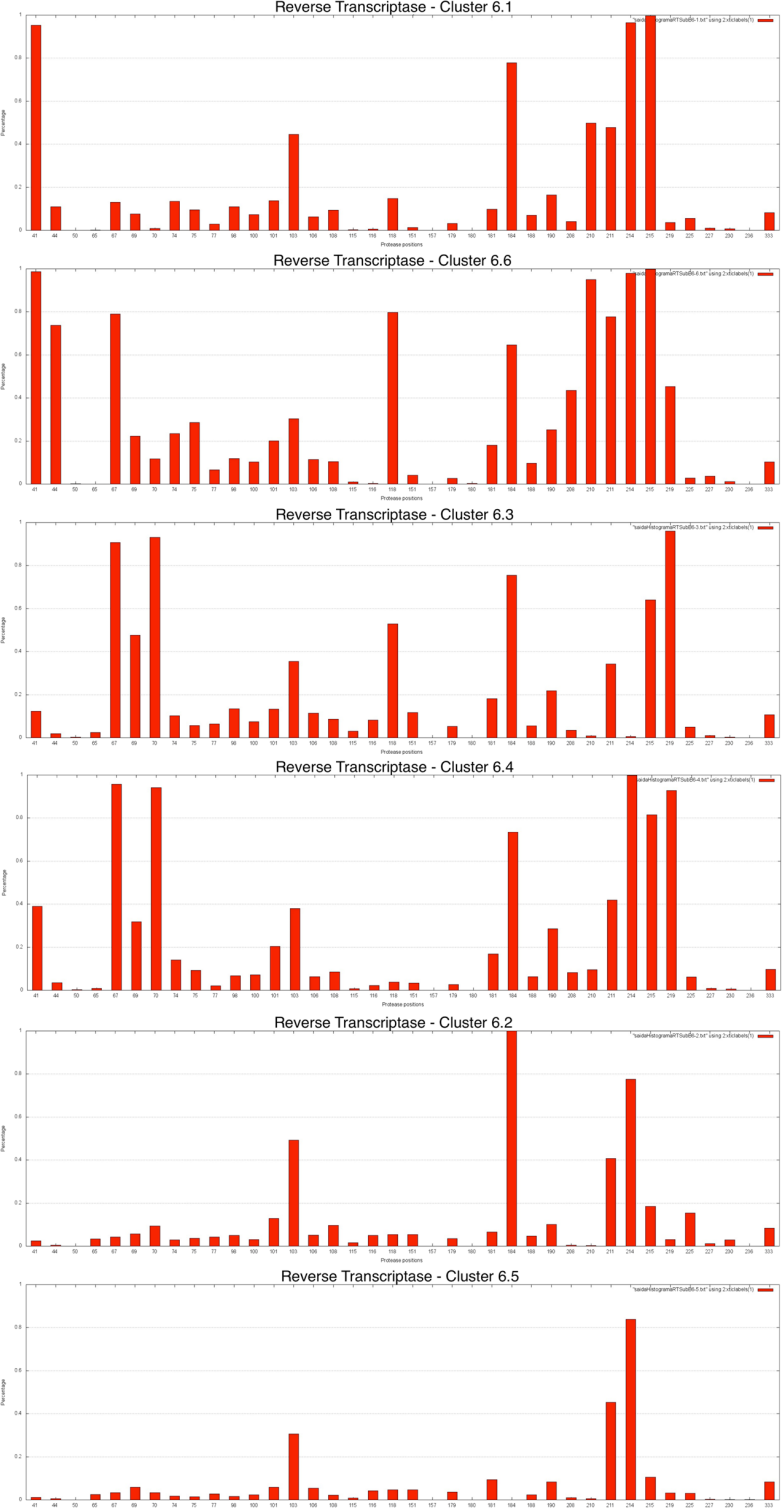
**Histogram showing the frequency of mutations in reverse transcriptase kmeans clusters.** Histograms containing the frequencies of mutations for each selected amino acid position in the reverse transcriptase for each of the six clusters in subtype B at *k*=6. Each histogram represents one cluster found by K-means for *k*=6 in the reverse transcriptase sequences. Each bar in the histogram represents a protein position and the percentage of sequences in the cluster that contain a mutation at that position.

To characterize the clusters, the histograms shown in Figures 6 and 7 for subtype B and k = 6, for protease and reverse transcriptase, respectively, were produced. These histograms display the percentage occurrence of mutations at each amino acid position for each cluster. The mutations that had higher percentages defined the clusters and determined which cluster the sequences belonged to. Those that had high frequencies in one cluster and low frequencies in the others enabled differentiation between the sequences and between the clusters. Additionally, the positions with higher frequencies of mutations in a cluster were those that occurred together more frequently, and their occurrences were considered as related.

**Figure 8:**
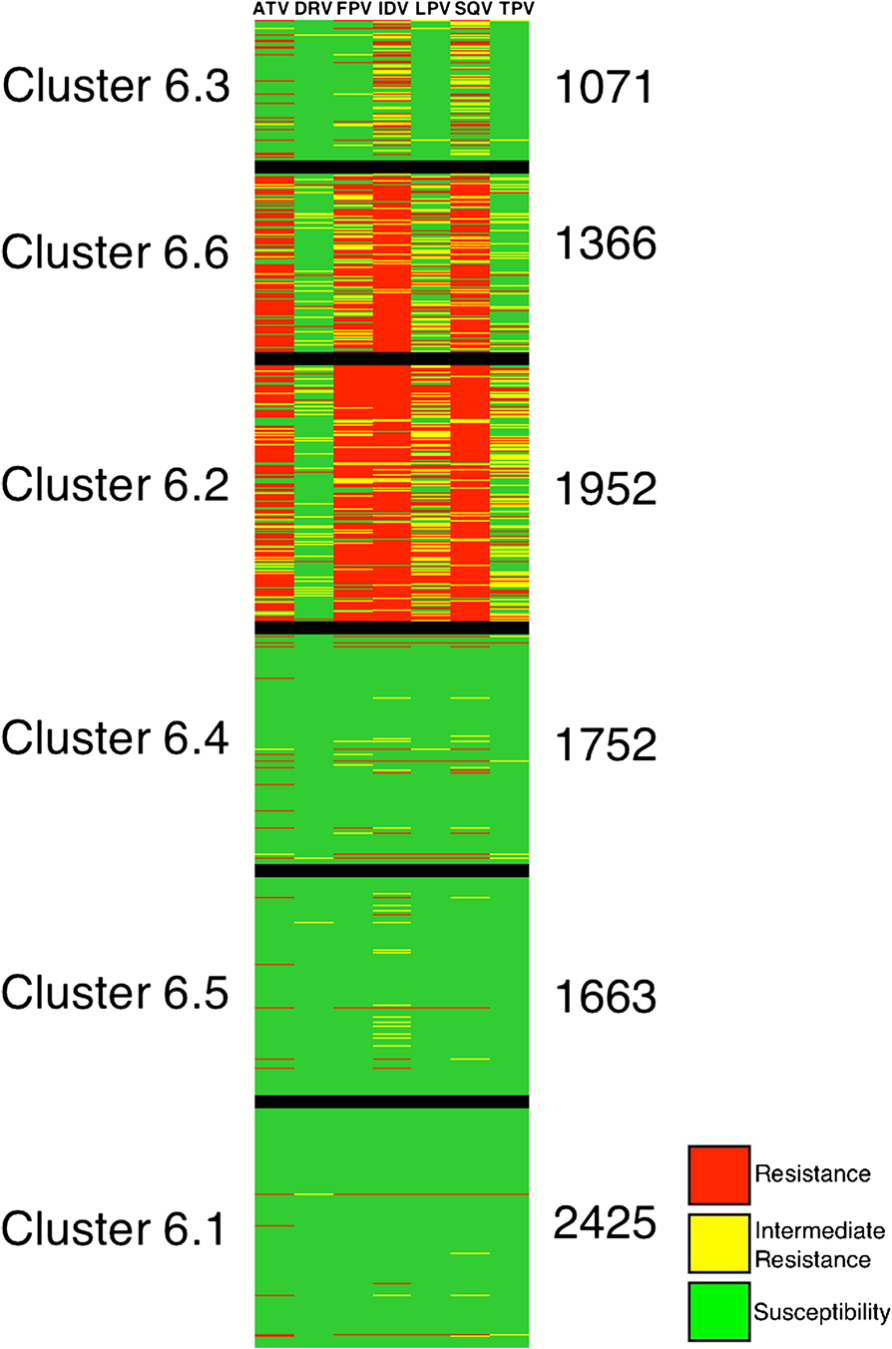
**Colored figure of the kmeans clusters for subtype B sequences of the HIV protease.** The figure displays the predictions of drug resistance from the Brazilian look-up table for each cluster. The columns in the colored figure represent the nine drugs selected (ATV/R, DRV/R, FPV/R, IDV/R, LPV/R, SQV/R and TPV/R, in that order) and the rows represent the protein sequences. Black lines delimit the classes. The number identifying each cluster is on the left and the number of the sequences in the cluster on the right.

**Figure 9:**
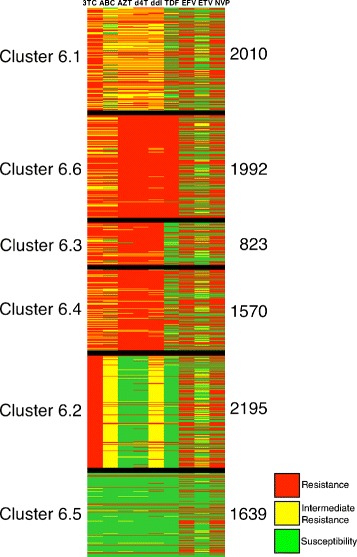
**Colored figure of the kmeans clusters for subtype B sequences of the HIV reverse transcriptase.** The columns in the colored figure represent the nine drugs selected (3TC, ABC, AZT, d4T, ddI, TDF, EFV, ETV and NVP, in that order) and the rows represent the protein sequences. Black lines delimit the classes. The number identifying each cluster is on the left and the number of the sequences in the cluster is on the right.

To compare the clusters with the predictions of drug resistance given by the rules in the Brazilian look-up table, colored images were created. The images from the protease clusters (see Figure 8 at k = 6) showed division of the sequences into groups that were sensitive to the majority of the drugs and other groups that were resistant to the majority of the drugs. However, the reverse transcriptase clusters showed different combinations of predictions for different clusters, with similar predictions for sequences in the same cluster and different predictions for sequences in different clusters (see Figure 9).

As seen in Figures 2 and 3, the clusters had different mutation profiles for the two proteins. K-means successfully distinguished the sequences and grouped them according to the different mutations, indicating that it is possible to obtain a classification for HIV protein sequences using clustering algorithms, according to the occurrences of the mutations.

The different occurrence patterns for the mutations are emphasized in Figures 6 and 7, which show the distinct percentages of mutations present at each protein position and at each cluster for subtype B. Some positions are important for the characterization and description of the clusters, such as positions 10, 82 and 90 of the protease, and 67, 70 and 219 of the reverse transcriptase.

Additionally, K-means was able to produce clusters that correlated with different predictions of drug resistance, especially for the reverse transcriptase (see Figure 9). The figures show that although clusters were found for both proteins, reverse transcriptase clusters display more patterns of prediction of drug resistance. As protease gene variation is higher than for reverse transcriptase gene in non-treated patients, the pathways for a strain to become resistance are more limited in reverse transcriptase as compared to the protease. Therefore, we believe that the constrains for variation in the reverse transcriptase gene facilitate the detection of the clusters.

The results for subtypes C and F are summarized in Tables 5 and 6. Tables 5 and 6 also attempt to summarize the clusters and depict the essential information that is necessary to understand and compare them. In these tables, the amino acid positions of the proteins are presented for positions where more than 50% of the sequences in the cluster had mutations.

**Table 5:** **Reverse transcriptase amino acid positions with mutations in at least 50% of the sequences by kmeans cluster**

		**Reverse transcriptase positions**										
**Cluster**	**Size**	**41**	**67**	**69**	**70**	**103**	**184**	**210**	**211**	**214**	**215**	**219**
Cluster B6.1	2010	X					X			X	X	
Cluster B6.2	2195						X			X		
Cluster B6.3	823		X		X		X				X	X
Cluster B6.4	1570		X		X		X			X	X	X
Cluster B6.5	1639									X		
Cluster B6.6	1992	X	X				X	X	X	X	X	
Cluster C6.1	89								X	X		
Cluster C6.2	60	X					X	X	X	X	X	
Cluster C6.3	37	X	X		X	X	X	X	X	X	X	X
Cluster C6.4	106					X	X		X	X		
Cluster C6.5	53		X	X	X		X		X	X	X	X
Cluster C6.6	59		X		X		X		X	X		X
Cluster F6.1	159	X					X		X	X	X	
Cluster F6.2	164								X	X		
Cluster F6.3	99	X	X				X	X	X	X	X	
Cluster F6.4	54						X		X			
Cluster F6.5	162		X		X		X		X	X	X	X
Cluster F6.6	94					X	X		X	X

**Table 6:** **Protease amino acid positions with mutations in at least 50% of the sequences by kmeans cluster**

		**Protease positions**																	
**Cluster**	**Size**	**10**	**13**	**15**	**20**	**30**	**35**	**36**	**41**	**46**	**54**	**62**	**63**	**71**	**82**	**88**	**89**	**90**	**93**
Cluster B6.1	2425												X						
Cluster B6.2	1952	X			X		X	X		X	X	X	X	X	X			X	
Cluster B6.3	1071		X			X	X	X					X	X		X			
Cluster B6.4	1752							X					X						
Cluster B6.5	1663												X						
Cluster B6.6	1366	X								X			X	X	X			X	X
Cluster C6.1	53			X	X		X	X				X	X				X	X	
Cluster C6.2	138			X				X									X		X
Cluster C6.3	114			X				X									X		X
Cluster C6.4	31		X	X	X	X	X	X					X			X	X		
Cluster C6.5	52	X		X	X		X	X			X		X	X	X		X	X	
Cluster C6.6	16	X	X	X				X					X					X	X
Cluster F6.1	89	X		X	X	X	X	X	X							X	X		
Cluster F6.2	70	X	X	X	X		X		X			X	X					X	
Cluster F6.3	81	X		X	X		X		X	X	X		X	X	X			X	X
Cluster F6.4	247			X			X	X	X								X		
Cluster F6.5	98	X		X	X		X	X	X	X	X		X		X		X		
Cluster F6.6	147			X			X		X								X		

Tables 5 and 6 show that for the different subtypes, the mutations that characterized some clusters were similar. The clusters from sequences of subtypes B, C and F were similar in terms of the positions in each cluster that had higher frequencies of mutations, excluding positions that occurred more frequently in a given subtype in this data set. For example, positions 15, 20, 36, 41, 69, 89 and 93 for subtype C in the protease; positions 15, 35, 36, 41 and 89 for subtype F in the protease; and position 211 for subtypes C and F in the reverse transcriptase. Moreover, the datasets for subtypes C and F were much smaller than the dataset for subtype B and thus might not represent all the variability in the subtypes. Subtype C was more different compared with subtypes B and F; however, there was still correspondence among the codons defining the clusters.

Correspondence among the clusters could be observed; for example, in protease clusters B6.2, C6.5 and F6.3, which had high percentages of sequences with mutations in positions 10, 54, 82 and 90 (as described in [10,16]) and clusters B6.3, C6.4 and F6.1 in positions 30 and 88 (as described in [7,9-12,23]). Reverse transcriptase clusters B6.3, B6.4, C6.5, C6.6 and F6.5 also showed correspondence and had high percentages of sequences with mutations in positions 67, 70 and 219 (as described in [6,9,25]) and clusters B6.6, C6.3 and F6.3 in positions 41, 67 and 210 (as described in [16]). Clusters B6.1, B6.4, B6.5, C6.2, C6.3, F6.4 and F6.6 from the protease and B6.2, B6.5, C6.1, C6.4, F6.2, F6.4 and F6.6 from the reverse transcriptase contained sequences with few mutations, and are probably susceptible to drugs.

Thus, the clusters suggested that mutations in codons 10, 54, 82 and 90, or in codons 30 and 88, in the protease are related and frequently occur together. In addition, mutations in codons 67, 70 and 219, or in codons 41, 67 and 210 in the reverse transcriptase frequently occur together. These patterns were also reported in previous studies [6,7,9-12,16,23,25] and will be important when investigating the genotype and phenotype (drug resistance) relationships and in designing new drugs.

## Conclusion

In this work, a new approach to analyzing HIV mutation data was presented. Current classification schemes are based on rule-based systems and look-up tables that comprise data from scientific studies. The proposed framework is based on a bitmap representation that extracts information from protease and reverse transcriptase sequences and provides information on the interactions among mutations.

A new visualization scheme inspired by microarray data analysis was proposed to better understand the clusters in the HIV domains. The images produced were useful for viewing and comparing the clusters with binary vectors and large volumes of data. In our study, the black and white figures indicated the occurrence and absence of mutations in sequences in each cluster, respectively, thus highlighting the differences between the clusters.

To represent the genetic variability of the virus in a different way from previous works, a large number of sequences and protein positions were used, along with three different HIV-1 subtypes. In the analysis, sequences were clustered, and the clusters were characterized according to the mutation patterns that they represented. The clusters were compared with those clusters revealed by previously published studies, and with the current knowledge of mutation patterns.

Along with the large number of sequences and protein positions, the application of a binary representation for the sequences helped to define a simple measure of similarity. The choice of K-means as the algorithm for mutation pattern searching rendered the method suitable for larger data sets because of its time complexity. The use of the binary image also allowed the analysis of large data sets, as the information in the data is visualized more easily, as is the characterization of the clusters and the mutation patterns.

K-means obtained clusters with similar sequences representing different mutation profiles, and the clusters showed that some mutations frequently occur together, which are important for defining the clusters and that are present in a large number of the sequences. These positions need to be taken into consideration when inferring drug resistance, because they affect a large number of patients.

Some interesting insights came from this clustering result. Notably, mutations in protease codons only produced clusters among non-B strains. Furthermore, as described previously, mutations at codons 89 and 90 in the protease do not cluster together [34], suggesting that methionine at positions 89 and 90 result in a protein structure that is not stable. Mutations at codons 30 and 90 may be selected by the protease inhibitor nelfinavir, but again, these two pairs of mutations do not appear together. It makes biological sense that once you have a replacement such as D30N, you will need a mutation N88D, because these two amino acids interact with each other in the protease protein [35]. However, it has been suggested that the pathway for resistance to nelfinavir will preferentially select the F30N complex among subtype B and exclusively the L90M complex among non-B subtypes [36]. However, we observed the D30N complex among clusters for subtypes B and F (Table 6). It is also interesting that major protease inhibitor mutations, such as in codons 46, 82 and 90, frequently form clusters (Table 6).

Pathways for resistance mutations are the pathways that viruses select for resistance mutations and this is closely related to cross-resistance. TAM 1 and TAM 2 are well-defined distinct pathways for resistance, but we speculate that these are merely initiating pathways because we observed clusters for the reverse transcriptase with between three and six TAMs, thus augmenting levels of resistance and cross resistance (Table 5). Interestingly, all clusters with resistance mutations show the 3TC-related mutation at codon 184 in the reverse transcriptase. When there is an antiretroviral treatment failure using non-nucleoside reverse transcriptase analogs, mutation at codon 103 will emerge in more than 50% of cases and 50% of these viruses will also harbor the mutation at codon 184 [37]. However, all clusters harboring 103 mutations will also be accompanied of 184 mutations, suggesting that real life virological failure is somehow different.

One interesting outcome from this cluster representation is their alleged relationships with previous exposure to specific antiretrovirals. In this sense, timing or the number of drug exposures, as well as the use of specific drugs, would suggest a specific selection of a cluster of mutations and imply possible resistance/cross resistance. The negative predictive value of a genotype result is low, meaning that the absence of a specific mutation or group of mutations does not mean that this mutation is not present in a minority population and is not present because of the selective pressure of current antiretrovirals being used. Therefore, the history of antiretroviral exposure and the projected profile of mutations can result in a more reliable future salvage therapy regimen.

Furthermore, protease inhibitors are designed according to the structure of the proteins; therefore, the clusters may help in designing future drugs for resistant strains.

In addition to antiretroviral resistance, understanding the mutation patterns is also useful in collaborative efforts to study of immune escape pathways and vaccine research. However, the HIV mutation patterns can confound the determination of the immune escape mechanisms [38] that are relevant to the vaccine research [39].

Our future work will include further validation of the clusters in the HIV domains and updating the current knowledge concerning mutations. We will also evaluate a recent approach to pattern recognition known as biclustering [40,41] for the protease and reverse transcriptase sequences. Biclustering algorithms seem to fit our purposes because they search for submatrices in the data matrix, following a determined pattern, and have been applied to large data sets, such as microarray data.
